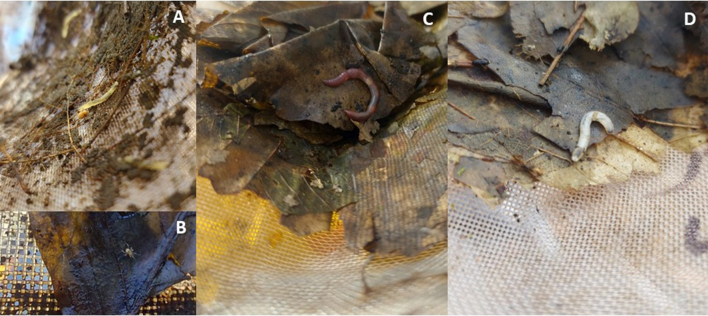# Large invertebrate decomposers contribute to faster leaf litter decomposition in *Fraxinus excelsior*-dominated habitats: Implications of ash dieback

**DOI:** 10.1016/j.heliyon.2024.e27228

**Published:** 2024-03-05

**Authors:** Cecilia A.L. Dahlsjö, Thomas Atkins, Yadvinder Malhi

**Affiliations:** a School of Geography and the Environment, South Parks Road, OX1 3QY, Oxford, Oxfordshire, UK; b Leverhulme Centre for Nature Recovery, University of Oxford, UK

**Keywords:** Ash dieback, Decomposition, *Fraxinusexcelsior*, Invertebrates, Leaf litter, Wytham woods

## Abstract

Leaf litter decomposition is a major component of nutrient cycling which depends on the quality and quantity of the leaf material. Ash trees (*Fraxinus excelsior*, decay time ∼ 0.4 years) are declining throughout Europe due to a fungal pathogen (*Hymenoscyphus fraxineus*), which is likely to alter biochemical cycling across the continent. The ecological impact of losing species with fast decomposing leaves is not well quantified. In this study we examine how decomposition of three leaf species with varying decomposition rates including ash, sycamore (*Acer pseudoplatanus,* decay time ∼ 1.4 years), and beech (*Fagus sylvatica,* decay time ∼ 6.8 years) differ in habitats with and without ash as the dominant overstorey species. Ten plots (40 m × 40 m) were set up in five locations representing ash dominated and non-ash dominated habitats. In each plot mesh bags (30 cm × 30 cm, 0.5 mm aperture) with a single leaf species (5 g) were used to include (large holes added) and exclude macrofauna invertebrates (with a focus on decomposer organisms such as earthworms, millipedes, and woodlice). The mesh bags were installed in October 2020 and retrieved without replacement at exponential intervals after 6, 12, 24 and 48 weeks. Total leaf mass loss was highest in the ash dominated habitat (ash dominated: 88.5%, non-ash dominated: 66.5%) where macrofauna were the main contributor (macrofauna: 96%, microorganisms/mesofauna: 4%). The difference between macrofauna vs microorganisms and mesofauna was less pronounced in the non-ash dominated habitat (macrofauna: 68%, microorganisms/mesofauna: 31%). Our results suggest that if ash dominated habitats are replaced by species such as sycamore, beech, and oak, the role of macrofauna decomposers will be reduced and leaf litter decomposition rates will decrease by 25%. These results provide important insights for future ash dieback management decisions.

## Highlights

• Leaf litter decomposition was 25% higher in ash dominated habitats.
• Macrofauna was the main driver of the difference between habitat types.
• Leaf litter quality and abundance influenced the role of macrofauna decomposers.
• Ash dieback is likely to lead to a reduction in leaf litter decomposition rates.

## 1 Introduction

Deciduous annual leaf production is the single largest contributor to annual net primary productivity (NPP) in temperate forests [1]. Leaf decomposition therefore plays a major role in top-soil nutrient dynamics [2,3]; although soil nutrient retention is closely linked to soil type [4]. Leaf decomposition rates are highly variable and depend on a range of factors, from climate and leaf quality to food web dynamics and the abundance and diversity of decomposer organisms [5]. While climate influences global trends in decomposition rates and leaf quality (lignin content, nitrogen (N) concentration, carbon and nitrogen (C/N) ratio), decomposer abundance and requirements have been shown to affect local scale variability [3,6].

Labile compounds, such as cellulose, decompose rapidly due to microorganisms’ ability to absorb them directly; however, recalcitrant compounds (e.g. lignin and chitin) are made up of larger molecules that cannot pass through the cell membrane [6]. These structures take a longer time to break down and so species whose leaves have high lignin content [7], such as beech (*Fagus* s*ylvatica*) and oak (*Quercus robur*) (decay time ∼ 6.8 years), have longer residence times compared with European ash (*Fraxinus excelsior*) (decay time ∼ 0.4 years) [8,9] whose leaves have low C/N ratios and low lignin content [6].

Macrofauna invertebrate decomposers (macrofauna invertebrates will be referred to as macrofauna from hereon) increase leaf decomposition both directly and indirectly by enhancing microbial activity and through direct consumption [6,10]. Macrofauna decomposers have been shown to preferentially select leaves based on their nutrient content and so the presence and preference of decomposer organisms may determine the decomposition rate of specific leaf species [5,11]. In Europe, the largest and most abundant macrofauna decomposers are earthworms (Oligochaeta), millipedes (Diplopoda), and woodlice (Isopoda). Vos et al. [11] showed that leaf decomposition increased when multiple leaf species were present; however, this effect has mainly been demonstrated when fast decomposing leaf species are added to mix of recalcitrant leaves [3,5]. Leaf litter quality, rather than the number of leaf species, therefore plays a larger role in leaf litter decomposition [5]. While the direct contact of fast decomposing leaves increase decomposition rates, the surrounding litter environment has been proposed to be of less importance [9].

The European ash tree is one of the latest tree species to be affected by an introduced non-native pathogen [12]. The disease (ash dieback) is caused by an invasive fungus (*Hymenoscyphus fraxineus*) which was first detected outside its native range in the early 1990s in Poland [13] and was confirmed in Britain in 2012 [14]. Across Europe the average ash mortality is estimated to be 70% [15], with higher mortality found in younger trees [16], which is expected to contribute to reduced levels of regeneration [12]. Limited ash regeneration is likely to lead to long lasting population declines of the European ash tree. Such declines will have a major effect on woodland ecology, as ash trees provide distinct habitats that replacement species cannot single-handedly restore [17]. In Britain, oak, beech, and sycamore (*Acer pseudoplatanus*) have been identified as some of the best replacements for ash due to similarities in species associations (oak and beech) and ecosystem functions (sycamore) [17]. Sycamore and beech have also been shown to be the most likely naturally regenerating species following ash dieback (i.e. most likely to replace ash) in southern and lowland Britain [17]. However, ash is unique, and while 74% of species associations would be represented by beech and oak, soil cation availability, soil pH and soil organic matter (SOM) content would only partially be met by planting species such as sycamore [17].

The impact of losing a single species with fast decomposing leaves is not well quantified; however, results from aquatic ecosystems suggest that it can have a major impact on leaf litter decomposition rates [18,19]. As species with more recalcitrant leaves are likely to replace the European ash, leaf residence times and leaf accumulation are thought to increase. Ash dieback is therefore expected to change the ecological landscape of European woodlands, altering the composition of the leaf litter, and resulting in an overall decline in decomposition rates. As we begin to see the outcomes of this disease across Europe, insights into the impacts of ash decline on leaf decomposition are needed to better understand the implications of ash dieback on nutrient cycling and wider ecosystem functioning. In this study we aim to examine how leaf litter decomposition of three leaf species with varying decomposition rates including ash (decay time ∼ 0.4 years), sycamore (decay time ∼ 1.4 years), and beech (decay time ∼ 6.8 years) [8,9] differ in habitats with and without European ash as the dominant overstorey species. Specifically, we ask.

(1) What are the relative roles of macrofauna (predominantly earthworms, millipedes, and woodlice) vs. microorganisms and mesofauna decomposers in the decomposition of ash, sycamore, and beech leaves?
(2) Is decomposition of different leaf species influenced by ash as a dominant overstorey species?
(3) How do the relative roles of macrofauna vs. microorganisms and mesofauna decomposers change during the decomposition of the three leaf species?

## 2 Materials and methods

### 2.1 Study site

This study was conducted in Wytham Woods, a temperate broadleaved woodland near Oxford in southern Britain (51° 46′ N, 001° 20’ W). Wytham Woods contain a mixture of old plantations, semi-natural woodland, and ancient woodland with the most common species being sycamore, beech, oak, ash, and hazel (*Corylus avellana*). The woods are situated on a hill with an elevation range between 56 m and 166 m (asl) (https://en-gb.topographic-map.com/maps/oh96/Wytham-Woods/, last visited on August 2, 2023). The mean annual temperature and rainfall are 10 °C and 726 mm, respectively [20]. Ash dieback was first detected in Wytham Woods in 2017 and is expected to have a major effect on the woodland ecosystem. However, at the time of sampling we found very low levels of ash mortality and canopy loss, and therefore the disease is unlikely to have influenced the results in this study.

### 2.2 Experimental design

**Fig. 1:**
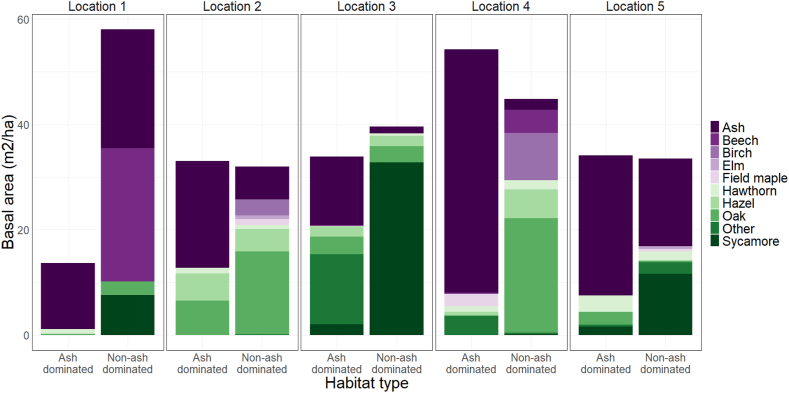
Tree species basal area in the plots separated by habitat type (ash dominated vs. non-ash dominated) and study location (1–5).

A leaf litter decomposition experiment was set-up in ten plots (40 m × 40 m) in a total of five locations (Fig. S1). Half of the plots were ash dominated and half were non-ash dominated with a mixture of chiefly sycamore, beech, oak, and hazel (Fig. 1). Two plots were situated in each of the five study locations at least 30 m apart, each representing a habitat type (ash dominated and non-ash dominated). The study locations were spaced at least a kilometre apart.

#### 2.2.1 Litterbag experiment

Mesh bags (30 cm × 30 cm) were constructed using 0.5 mm aperture mesh, designed to exclude British macrofauna decomposers (mainly anecic and epigeic earthworms, woodlice, and millipedes), and closed using stainless steel staples. Half of the bags were kept intact while 18 holes (1 cm diameter) were made in the remaining half to allow macrofauna to enter. Mesofauna and macrofauna overlap in size [21], however, this overlap by definition refers to body length rather than width. The latter is relevant when considering mesh aperture as this will determine which taxa can enter the experimental bags. While most litterbag studies use 1 mm mesh to exclude macrofauna, we found that when juveniles and body widths were considered, 0.5 mm mesh was more effective at excluding macrofauna that were of interest in this study (earthworms, millipedes, and woodlice) while including the majority of the mesofauna. During the spring mesh bag sampling, fauna that were encountered inside the exclusion mesh bags were recorded and photographed to ensure that the exclusion of macrofauna was working as planned. Samples were discarded if earthworms, millipedes, or woodlice were present within the exclusion bags.

Leaves from ash, sycamore, and beech were collected from trees and fallen branches in September 2020 and dried to constant weight at 70 °C. Five grams of dried leaves from each species were placed into separate mesh bags. The mesh bags were placed in the plots in October 2020 to coincide with naturally occurring leaf fall. In each plot the bags were placed in three clusters in a diagonal pattern (72 bags per plot, 720 bags in total) (Fig. S2). Bag retrieval took place without replacement at exponential intervals after 6, 12, 24 and 48 weeks.

During sampling the leaves inside the bags were extracted and placed into paper bags after which they were dried to constant weight at 70 °C and weighed to the nearest two decimals. In this paper “decomposition” refers to leaf mass loss, however, we acknowledge that the loss of leaf material may also be due to leaf fragmentation and that an accumulation of micro particles may influence the final weight. While we have taken steps to minimise the latter by brushing the leaves and removing visible material, the accumulation of micro particles should be consistent across habitat types and therefore have a minor impact on the results. A proportion (9 macrofauna inclusion bags each from ash and non-ash dominated habitats from a separate study location each time) of the leaves were weighed before drying to record the moisture content. A surplus of macrofauna inclusion bags (27) was carried to the plots to mimic installation and the leaves were later dried and weighed to examine the impact of handling. These included six bags with ash leaves, nine with beech leaves and 12 with sycamore leaves.

#### 2.2.2 Invertebrate extraction

Macrofauna were collected from the leaf litter and the soil in each plot every quarter (February, May, August, November). One quadrat per plot was sampled due to the destructive sampling method and to reduce disturbance (20 quadrats per habitat type across the experimental period). In each quadrat (1 m^2^) the leaf litter was collected and placed in a litter sieve. All invertebrates were gathered from the sieved material by hand sorting. Invertebrates from a soil pit in the centre of each quadrat (25 cm × 25 cm × 10 cm depth) were also hand sorted. Individuals were placed in 85% ethanol and identified to order [[22], [23], [24], [25]]. Macrofauna that predominantly feed on leaf litter were used in the analysis including earthworms (epigeic and anecic), millipedes, and woodlice.

#### 2.2.3 Tree census, environmental variables, and leaf fall

All trees in the study plots were identified to species and the diameter at breast height (dbh, 1.3 m) was recorded for each tree. These measurements were used to determine tree densities as well as the basal area for each species. Temperature (12 cm above ground) and soil moisture (0–15 cm depth) were measured using five TOMST data loggers in each plot which recorded data at 15 min intervals. Leaf fall in each plot was estimated by weighing the fallen leaves from six litter traps (0.3 m^2^–0.4 m^2^) per plot. The leaf litter was collected once a month (twice a month in autumn) then dried to constant weight at 70 °C.

### 2.3 Data analyses

#### 2.3.1 Litterbag experiment

The data were analysed using R (version 2021.09.0) with associated packages. Icons used in the figures were sought from PhyloPic (http://phylopic.org). The mean temperature and volumetric moisture for the sampling period in the two habitat types were calculated using daily averages and the statistical differences were examined using Welch two sample t-tests. Tree density in each plot was calculated as the number of trees per hectare and tree basal area was calculated using equation (1).

(1) $$basal\;area\;\left(m^{2}\right)=\pi r^{2}$$

where *r* is the radius of the tree trunk at breast height. The basal areas were summed for each species and plot and converted to m^2^/ha.

The dry weight of the leaves in the mesh bags was calculated as the percentage of remaining leaves based on an initial starting weight of 5 g. Outliers of more than three standard deviations (14 samples in total) were removed. The time between retrievals was logged using natural logarithms as samples were collected at exponential intervals (6, 12, 24 and 48 weeks after installation). Correlations between the collected variables were examined using [cor.test] and highly correlated variables (>70% correlation) were not modelled together to avoid autocorrelation. The data were examined using a mixed linear effects model [lmer] with bag location as the random factor. The minimal adequate model was determined using Akaike information criterion (AIC) and the statistical differences were explored using ANOVA (Table 1).

**Table 1:** Full and minimal adequate model with *leaf mass remaining* at each retrieval as the dependent variable.

Terms	numDF	denDF	*F*-value	*P*-value
*Full modell*				
Mesh	1	54.0	108.5	1.6E-14*
ln(retrievals)	1	641.2	862.7	8.5E-121*
Leaf species	2	641.4	404.0	2.8E-114*
Habitat type	1	56.2	13.0	0.001*
Tree density (tree/ha)	1	53.9	0.3	0.6
Tree basal area (m2/ha)	1	55.0	0.3	0.6
Soil moisture	1	632.8	6.3	0.01*
*Minimal adequate model*				
Mesh	1	57.0	103.2	2.1E-14*
ln(retrievals)	1	642.7	864.3	4.8E-121*
Leaf species	2	643.0	403.4	3.0E-114*
Habitat type	1	57.0	20.6	3.0E-05*

* = significant interactions.

The proportional contribution of macrofauna to leaf litter decomposition was calculated using equation (2).

(2) $$Macrofauna\;contribution\;\left(\%\right)=\frac{M_{ex}-M_{tot}}{M_{ex}}*100$$

where *M*_*ex*_ is the mass of the leaves in mesh bags excluding macrofauna (typically slowest decomposition) and *M*_*tot*_ is the mass of the leaves in mesh bags including macrofauna i.e., decomposition by all decomposer organisms. The calculations were paired so that mesh bags adjacent to each other in the experimental set-up were compared. This was done to reduce noise due to spatial variation. The microbial and mesofauna contribution was calculated as the remaining percent. Outliers of more than three standard deviations from the mean were removed (32 samples in total).

The decomposition data and the data on the proportional contribution of macrofauna vs. microorganisms and mesofauna were plotted across the sampling retrievals to visualise differences among habitat types and leaf species over time. For each leaf species in the two habitats one-way-ANOVA was used to examine the statistical differences between each paired interaction (mesh type or proportional contribution) at each retrieval. Hedges' g effect size was thereafter calculated ([cohen.d] with [hedges.correction] in R) for each interaction. Hedges' g was preferred over Cohen's d as it provides a correction for small sample size bias (n = 15). Effect sizes were plotted to visualise the relationships and the significance of the change of the contributions over time.

The decomposition constant *k* was calculated using equation (3).

(3) $$k=\frac{-\ln\frac{M_{1}}{M_{0}}}{t}$$

where *M*_*1*_ is the leaf mass at the end of the experiment, *M*_*0*_ is the initial leaf weight (5 g), and *t* is the time in months. When leaves had decomposed completely (mass = 0) at the end of the experiment (11 months) the leaf mass from the previous retrievals was used. The difference between *k* when macrofauna was included and excluded was used to estimate *k* for macrofauna contribution. The impact of decomposer organisms on the decomposition (*k*) of leaf species in the different habitat types was examined using one-way-ANOVA, while Hedges g was used to calculate the effect sizes of paired interactions.

Handling error was examined using linear models for each leaf species, while the effect of leaf moisture was examined using mixed linear effects models for each leaf species with mesh bag location as the random factor. Statistical differences were examined using ANOVA.

#### 2.3.2 Invertebrate extraction

Macrofauna biomass, gathered from the invertebrate extraction sampling, was used to calculate metabolic rates (Jh) and daily food intake. This was done to examine the difference between leaf resource availability, leaf litter decomposition, and the energy demands of the leaf litter feeding invertebrates. Equations (4), (5)) were used to calculate metabolic rates based on models for earthworms and terrestrial arthropods, respectively [26]. The latter (equation (5)) was used to calculate the metabolic rate of both millipedes and woodlice.

(4) $$\ln\;(metabolic\;rate\;(J/h/ha))=5.70+0.71*\ln(mass\;per\;ha)-0.25*\frac{1}{k(temp+273.15)}$$

(5) $$\ln\;(metabolic\;rate\;\left(J/h/ha)\right)=13.98+0.91*\ln\left(mass\;per\;ha\right)-0.48*\frac{1}{k\left(temp+273.15\right)}$$

where *mass* per *ha* is milligrams per hectare, *k* is a constant (0.0000862), and *temp* is the temperature in Kelvin.

The metabolic rate was converted to kJ/ha/day and used to calculate the daily food intake using equation (6).

(6) $$Daily\;food\;intake\;\left(dry\;weight\right)=\frac{Daily\;metabolic\;rate}{Food\;energy*Assimilation\;efficiency}$$

where the daily food intake is in wet g/ha/day, daily metabolic rate is in kJ/ha/day, food energy is the energy in the leaves (20.7 kJ/g, Crocker et al. [27]), and assimilation efficiency is the ratio of the food absorbed by the body compared with the amount of food that is ingested. The latter differs among taxa and here we use 0.05 for earthworms, 0.13 for woodlice and 0.20 for millipedes based on the values reported in Schaefer [28].

#### 2.3.3 Leaf litter traps

Leaf fall was summed per litter trap per year and averaged at the plot level. The leaf fall data were then converted to leaf fall per ha per year and averaged across the plots to obtain a habitat mean.

## 3 Results

### 3.1 Litterbag experiment

In total, 720 litter bags were installed in the two habitat types of which 719 bags were successfully retrieved. Mesofauna were encountered in the majority of macrofauna exclusion bags while macrofauna (solely epigeic earthworms) were present in very few bags (Fig. S3). As larger Enchytraeidae were present, this suggests that the mesh size of 0.5 mm was large enough to allow the vast majority of mesofauna to enter including collembola, mites, and smaller insects. The handling of mesh bags affected sycamore and beech leaves significantly more than bags with ash leaves with an average loss of 8.2% for sycamore, 6.5% for beech and 0.5% for ash (chi-square means; ash – sycamore, t = 4.9, p < 0.001; ash – beech, t = 2.8, p < 0.05; beech – sycamore, t = −2.4, p > 0.05, Fig. S4).

#### 3.1.1 Leaf litter decomposition rates

Leaf litter decomposition was significantly associated with leaf species, mesh type, habitat type, and the logged retrieval dates (ln) (Table 1). When macrofauna were included, ash leaves decomposed faster (ash dominated habitat k = 0.5 ± 0.04, non-ash dominated habitat k = 0.4 ± 0.04) than sycamore (ash dominated habitat k = 0.3 ± 0.04, non-ash dominated habitat k = 0.2 ± 0.04) and beech (ash dominated habitat k = 0.2 ± 0.04, non-ash dominated habitat k = 0.1 ± 0.04). However, there was no significant difference between the decomposition constants *k* for sycamore and beech (chi-square means, ash-sycamore: t = 3.6, *p* < 0.01, ash-beech: t = 5.7, *p* < 0.0001, sycamore-beech, t = 2.0, *p* > 0.05).

**Fig. 2:**
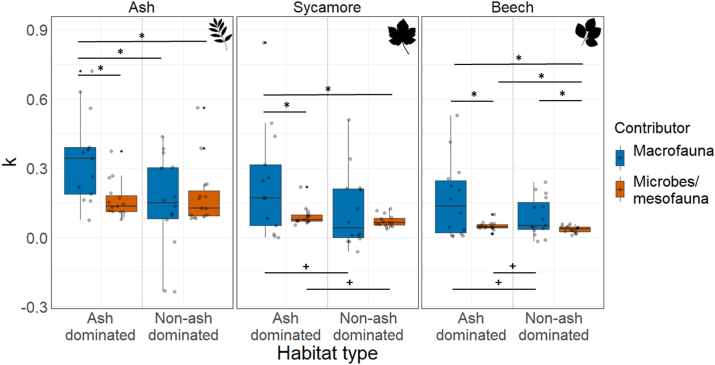
Decomposition constant (*k*) of ash, sycamore, and beech in the ash dominated and non-ash dominated habitats separated into macrofauna (blue) vs. microbial and mesofauna (orange) contribution. * represents significant (p > 0.05) differences between the paired bars indicated by the lines, while + represent interactions with high to medium effect sizes (Hedges g > 0.5). (For interpretation of the references to colour in this figure legend, the reader is referred to the Web version of this article.)

For ash leaves, macrofauna contributed significantly more to the decomposition rate (*k*) in the ash dominated habitat than in the non-ash dominated habitat (Fig. 2). In both habitat types, macrofauna also contributed significantly more to ash leaf decomposition than microorganisms and mesofauna. For sycamore, macrofauna contributed significantly more to leaf decomposition than microorganisms and mesofauna in both habitat types. However, leaf decomposition by macrofauna was not significantly different between habitat types, although the effect size was medium (Hedges' g; effect size = 0.6) (Fig. 2). Macrofauna contributed more to the decomposition rates of beech leaves in the ash dominated habitat than microorganisms and mesofauna. No significant difference between habitats was found for macrofauna driven beech decomposition, although the effect size was medium (Hedges’ g; effect size = 0.6) (Fig. 2). The contribution of microorganisms and mesofauna to beech leaf decomposition was significantly different between habitat types with higher decomposition in the ash dominated habitat (Fig. 2).

#### 3.1.2 Temporal variation in leaf mass loss among habitat types, leaf species, and mesh bags

**Fig. 3:**
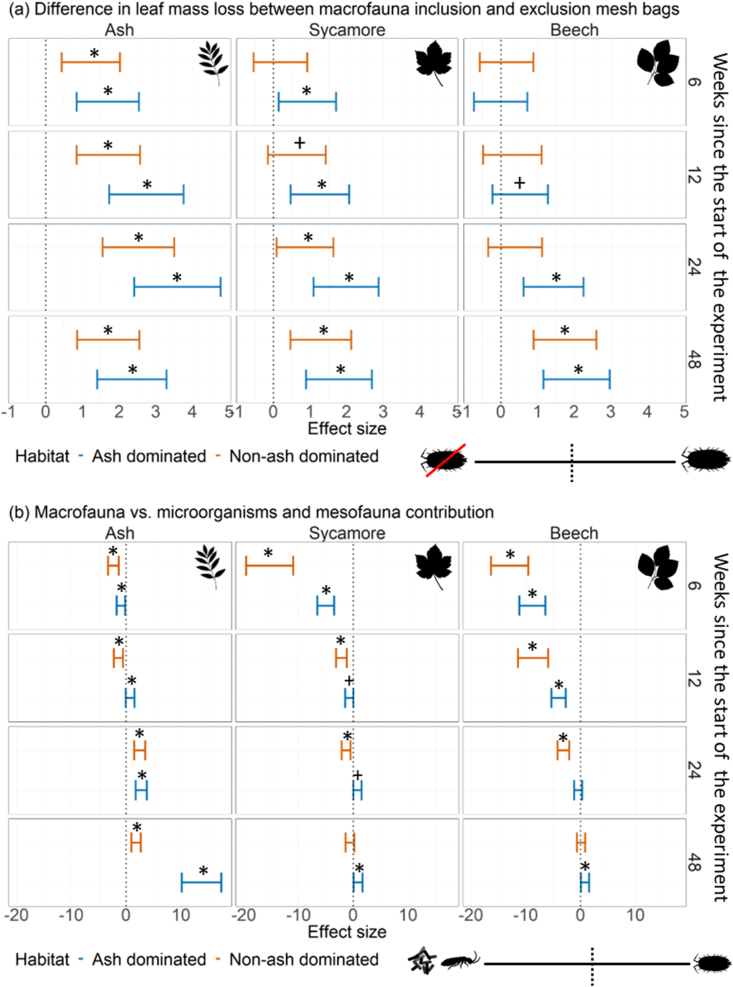
Effect sizes (Hedge's G) (±95% confidence interval) for (A) differences between macrofauna inclusion and exclusion bags and (B) the contribution of macrofauna vs. microorganisms and mesofauna to decomposition of ash, sycamore, and beech leaf litter. In (A), if the effect size is positive, leaf decomposition in the macrofauna inclusion bags was higher than in the exclusion bags. In (B), if the effect size is positive, macrofauna contributed more to decomposition than microorganisms and mesofauna (B). * indicates large effect sizes (<−0.8 or >0.8) and + indicates medium effect sizes (−0.8 to −0.5 or 0.5 to 0.8).

The mass loss of ash leaves was higher in the macrofauna inclusion bags in both the ash dominated and non-ash dominated habitats at all sampling occasions (Hedges' g; effect size >0.8) (Fig. 3a). In the ash dominated habitat mass loss of sycamore leaves was similarly higher in the macrofauna inclusion bags at all sampling occasions (Hedges' g; effect size >0.8). For beech leaves in the ash dominated habitat the mass loss between macrofauna inclusion and exclusion bags showed a medium effect size from week 12 of the experiment (Hedges' g; week 12: effect size = 0.5, week 24 onwards: effect size >0.8) (Fig. 3a). There was no difference between macrofauna inclusion and exclusion bags for leaf decomposition in the non-ash dominated habitat until week 12 of the experiment for sycamore (Hedges' g; week 12: effect size = 0.6, week 24 onwards: effect size >0.8) and until week 48 of the experiment for beech (Hedges’ g; effect size >0.8) (Fig. 3a).

When comparing the roles of macrofauna vs microorganisms and mesofauna the impact of the latter played a major role for leaf mass loss of all species in both ash dominated, and non-ash dominated habitats in the first six weeks of the experiment (Hedges' g; effect size >0.8) (Fig. 3b). By week 12 macrofauna played a larger role than microorganisms and mesofauna for mass loss of ash leaves in the ash dominated habitat (Hedges' g; week 12 onwards: effect size >0.8) followed by sycamore leaves by week 24 (Hedges' g; week 24 onwards: effect size ≥0.8) and beech leaves by week 48 (Hedges' g; effect size >0.8) (Fig. 3b). In the non-ash dominated habitat ash leaf mass loss was predominantly driven by macrofauna from week 24 onwards (Hedges' g; effect size >0.8), while microorganisms and mesofauna played the largest role in leaf mass loss of sycamore and beech for the duration of the experiment (Hedges' g; effect size < −0.8), although there was no difference between contributors at week 48 (Hedges’ g; effect size <0.1) (Fig. 3b).

### 3.2 Invertebrate food intake and leaf fall

Leaf fall was higher in the non-ash dominated habitat, although not significantly so (Table 2). There was not a significant difference in the total amount of decomposed leaf litter between habitat types, however, there was a difference in the role of the decomposers. Macrofauna contribution was higher in the ash dominated habitat, while microorganism and mesofauna contributions were significantly higher in the non-ash dominated habitats (Table 2). The annual average leaf mass decomposed by macrofauna was many times higher than the estimated food intake by the three main taxa (earthworms, millipedes, and woodlice) based on biomass measurements (Table 2). While there was no difference in earthworm food intake between the habitat types, it was higher in the ash dominated habitat for millipedes and woodlice (Table 2). There was no significant seasonal difference in food intake for either of the macrofauna decomposers, although effect sizes were medium to large in November and February when leaf decomposition peaked (Fig. S5).

**Table 2:** Annual dry leaf fall, leaf decomposition, contributions to decomposition by macrofauna, and microorganisms and mesofauna, and food intake for ash dominated and non-ash dominated habitats. Numbers in brackets indicate the proportion contribution towards the total decomposed leaves or macrofauna food intake. Positive effect sizes indicate that the values were higher in the ash dominated habitat, while negative effect sizes indicate higher values in the non-ash dominated habitat.

Variables (kg/ha/year)	Ash habitat		Non-ash habitat				
	mean	se	mean	se	*F*	*P*	Effect size
Leaf fall (dry weight)	2168.5	293.0	2787.1	850.5	0.5	>0.05	−0.4
*Decomposed leaves*							
Total decomposed	1919.8	280.1	1853.0	591.1	0.01	>0.05	0.06
Macrofauna	1839.7 (96%)	273.0	1270.4 (68%)	474.8	1.1	>0.05	**0.6**
Microorganisms/mesofauna	80.0 (4%)	40.8	582.6 (31%)	168.4	8.4	<0.05*	**−1.7**
*Macrofauna food intake*							
Total food intake	26.6	4.5	22.5	8.3	0.2	>0.05	0.3
Earthworm (epigeic and anecic)	20.5 (77%)	3.2	20.1 (89%)	8.3	0.002	>0.05	0.03
Millipede	2.1 (8%)	1.2	0.3 (1%)	0.2	1.9	>0.05	**0.8**
Woodlouse	3.9 (15%)	2.1	1.8 (8%)	0.5	1.0	>0.05	**0.6**

**Bold** = medium (0.5–0.8) to large (>0.8) effect sizes.

* = significant differences between habitat types.

### 3.3 Tree census and environmental variables

During the experiment (October 2020–September 2021) soil moisture across the ash dominated habitat (mean ± SE, 57.7 ± 0.2%) was significantly higher than in the non-ash dominated habitat (52.4 ± 0.3%) (t.test, t = 10.7, p > 0.0001), while ambient temperature was not significantly different (*t*-test, *t* = −1.0, *p* > 0.05, ash dominated 9.2 ± 0.1 °C, non-ash dominated 9.4 ± 0.1 °C). Leaf moisture in the mesh bags was not significantly different among leaf species (ANOVA(lmer), *F* = 0.7, *p* > 0.05) or habitat types (ANOVA(lmer), *F* = 0.2, *p* > 0.05) (Fig. S6). The average tree basal area and tree density varied among plots, however, were not significantly different between the habitat types (ash dominated: mean ± SE basal area 33.8 ± 6.4 m2/ha and tree density 771.3 ± 137.8 trees/ha; non-ash dominated: basal area 41.6 ± 4.7 m2/ha and tree density 1066.3 ± 82.3 trees/ha) (basal area: ANOVA, *F* = 1.0, *p* > 0.05, tree density: ANOVA, *F* = 3.4, *p* > 0.05). The tree species composition varied by location (Fig. 1) but on average ash trees had the highest basal area (23.8 ± 6.2 m^2^/ha, 70%) in the ash dominated habitat. The non-ash dominated habitats were mainly comprised of beech (14.8 ± 10.4 m^2^/ha, 24.7%), sycamore (13.5 ± 9.9 m^2^/ha, 22.5%), and oak (10.8 ± 4.7 m^2^/ha, 18.0%). While the ash basal area was sometimes higher in the non-ash dominated plots than in the ash dominated plots, the dominance of ash as the overstorey species was consistent with habitat type.

## 4 Discussion

### 4.1 Drivers of leaf mass loss

In agreement with other studies [9], ash leaves decomposed the fastest followed by sycamore and then beech. The handling error was higher for sycamore (4.5%) and beech (8.2%) than for ash (0.3%) which contributed to a slight overestimation of the initial mass loss and a conservative measure of the difference in decomposition between the leaf species. Additionally, the fine mesh may have resulted in an accumulation of microparticles over time, although this would have been the case across all mesh bags with little effect on the differences between habitat types. Moisture has been shown to play a major role in influencing leaf decomposition at the local scale [6,10] and while the ash dominated plots had higher soil moisture than the non-ash dominated plots, there was no significant difference in the leaf water content in the mesh bags. While plot level soil moisture may have increased macrofauna decomposer activity [29] this was not reflected in our measurements of macrofauna biomass and food intake.

Decomposition of all leaf species was higher in the ash dominated habitat than in the non-ash dominated habitat but only when macrofauna were included. Additionally, macrofauna played a larger role in the decomposition of the fast-decomposing leaf species (ash) than the more recalcitrant species (sycamore and beech) in both habitat types. The role of macrofauna was larger in the ash dominated habitat than in the non-ash dominated habitat where they contributed 96% to leaf decomposition while microorganisms and mesofauna contributed 4%. In the non-ash dominated habitat macrofauna contributed 68% to leaf decomposition, while microorganisms and mesofauna contributed 31%.

While species composition of leaf litter on the ground has been shown to have a non-significant effect on leaf decomposition [9] we believe that the environment that ash trees create as a dominant overstorey species is one of the major drivers of the strong macrofauna signal in this study. The two habitat types differed in tree species composition, density, and leaf litter quality. We found that 88.5% of the fallen leaves in the ash dominated habitat were decomposed annually compared with 66.5% in the non-ash dominated habitat, suggetsing that 11.5% and 33.5% of leaf fall accummulates in the ash dominated and non-ash dominated habitats respectively. In practice, the difference in leaf accummulation creates two very different habitats, one with a negligable leaf litter layer and one with a thick leaf litter layer. The ash dominated habitat provides a high quality but low abundance leaf litter resource, while the non-ash dominated habitat provides a low quality but high abundance leaf litter resource.

In agreement with other studies [6], microorganisms and mesofauna contributed more to leaf decomposition than the macrofauna in the first six weeks of the experiment. After the initial stage of decomposition, the contribution of the different decomposer organisms changed with more emphasis on macrofauna led decomposition at the end of the experiment. We found a distinct lag in the shift from microbial and mesofaunal decomposition to macrofauna driven decomposition among the leaf species. Macrofauna became the dominant driver of ash decomposition sooner than for sycamore and beech leaves. The macrofauna shift from ash to sycamore and beech was distinct for the ash dominated habitat suggesting that macrofauna target the ash leaves and then move on to more recalcitrant leaves when ash is no longer available. This signal was much less obvious in the non-ash dominated habitat, in which macrofauna decomposition was never significantly higher than the microbal and mesofauna decomposition for sycamore and beech.

### 4.2 Macrofauna food intake and leaf litter decomposition

Macrofauna were the main drivers of the differences in leaf litter decomposition between habitat types in this study. However, the three main macrofauna taxa (woodlice, millipedes, and earthworms) had low food intake relative to the proportion of leaves that were decomposed in the mesh bags on the ground. The discrepancy between the decomposed leaves and estimated food intake suggests that other macrofauna (e.g. molluscs, fly larvae) play a large role in leaf decomposition or that the sampling of macrofauna biomass was incomplete, due to the patchy distribution of the sampled taxa.

Earthworm food intake did not vary between the habitat types, but the food intake of woodlice and millipedes was higher in the ash dominated habitat than in the non-ash dominated habitat. The difference between the habitat types also appeared to be seasonal with larger differences in the months when leaves fell (November) and when microclimate conditions were favourable (November and February, wetter and cooler) [30]. This pattern suggests that the sudden spike in leaf litter availability in the ash dominated habitat increased macrofauna decomposer activity; however, the sampling method may have failed to pick this signal up sufficiently due to low replication and the highly mobile and patchy distribution of macrofauna. More targeted sampling that includes higher temporal and spatial replication of macrofauna is therefore needed to fully understand this relationship.

Millipedes and woodlice have been shown to be proportionally more important for the decomposition of recalcitrant leaf species [5,11], while earthworms play a larger role in the decomposition of fast decomposing leaves [5]. Leaf nutrient composition also influences how attractive leaves are to macrofauna decomposers based on their requirements for certain elements. It has been demonstrated that decomposition rates decline as C/N ratio and lignin content increase [6,7,31]. Earthworm decomposition has been found to be positively associated with leaf calcium (Ca) content, while manganese (Mn) content in leaves has been shown to increase fungal decomposition rates [32]. Ash leaves contain 33% higher Ca levels than beech leaves and 35% lower C/N ratio, while beech leaves contain 83% higher Mn levels [33]. These data suggest that earthworms are important for ash leaf decomposition, while fungi, millipedes and woodlice are important for the decomposition of more recalcitrant leaves. The higher biomass of millipedes and woodlice in the ash dominated habitat may explain the higher decomposition of sycamore and beech compared with the non-ash dominated habitat.

### 4.3 Long term effects of ash dieback on leaf decomposition

Based on the results in this study, in habitats where ash is the dominant overstorey species, leaf decomposition is expected to decrease by 25% following ash dieback if ash trees are replaced by species with more recalcitrant leaves. The average estimated ash mortality following ash dieback across Europe is 70% [15] suggesting that around 30% of the ash canopy is likely to survive. Our non-ash dominated habitat represents this scenario well with an average of 20% of ash in the canopy. Our results suggest that the absolute basal area or density of ash trees have a lesser impact on decomposition rates than the proportional dominance of ash as the overstorey species. This is likely to be due to the high quality but low abundance leaf litter resource that ash dominated habitats provide - thereby attracting macrofauna that further promote decomposition of other more recalcitrant leaf species. Our results also indicate that a reduction in ash leaf litter will change the role of the decomposer organisms such that the proportion of macrofauna driven decomposition will be reduced.

The decrease in leaf decomposition rates following ash dieback is expected to lead to a reduction in nutrient cycling. The topsoil (0–10 cm) is reactive to leaf decomposition and tends to contain higher levels of plant available cations in areas with high ash cover (Dahlsjö et al. unpublished data). The naturally sparse ash canopy also enables greater light penetration and so ash dominated habitats are more likely to have denser and more diverse vegetation cover compared with non-ash dominated habitats [34]. However, the thicker leaf litter layer and dense canopy in non-ash dominated habitats may result in a more buffered ecosystem with less extreme fluctuations in temperature and soil moisture [35] which may have an impact on seedling establishment [36]. The presence of a constant leaf litter layer may also provide an important habitat for some invertebrate taxa such as beetles [37].

### 4.4 Conclusion

In this study we found that leaf decomposition rates were higher in ash dominated habitats, and that this was driven by macrofauna decomposers. The relative importance of macrofauna is likely to be due to the nutrient rich leaf litter that ash dominated habitats provide. Therefore competition for ash leaf litter is relatively high, resulting in an increase in leaf decomposition of the adjacent more recalcitrant species once the ash resource has been depleted. Conversely, the low quality but high abundance resource pool in the non-ash dominated habitat resulted in slower decomposition of leaf litter with a higher proportion of the decomposition conducted by microorganisms and mesofauna. In the long-term, ash dieback could lead to a wide-ranging reduction in leaf decomposition which will have consequences for nutrient cycling. Our results will help inform ash dieback related management and provide better understanding of the role that ash trees play in influencing leaf decomposition and nutrient cycling.

## Data availability statement

Data associated with this study will be made available on request.

## Ethics statement

Review and/or approval by an ethics committee was not needed for this study because no human or animal subjects were used.

Informed consent was not required for this study because this study did not include humans or animals.

## CRediT authorship contribution statement

**Cecilia A.L. Dahlsjö:** Writing – review & editing, Writing – original draft, Visualization, Resources, Project administration, Methodology, Investigation, Funding acquisition, Formal analysis, Data curation, Conceptualization. **Thomas Atkins:** Writing – review & editing, Writing – original draft, Methodology. **Yadvinder Malhi:** Writing – original draft, Supervision, Project administration, Conceptualization.

## Declaration of competing interest

The authors declare the following financial interests/personal relationships which may be considered as potential competing interests: Cecilia Dahlsjo and Yadvinder Malhi reports financial support was provided by National Environment Research Council. If there are other authors, they declare that they have no known competing financial interests or personal relationships that could have appeared to influence the work reported in this paper.

## Figures and Tables

**figs1:**