# Supplementary material for: Large invertebrate decomposers contribute to faster leaf litter decomposition in Fraxinus excelsior-dominated habitats: Implications of ash dieback
Source: Heliyon. 2024 Mar 5;10(5):e27228. doi: 10.1016/j.heliyon.2024.e27228 (PMC10943353; doi:10.1016/j.heliyon.2024.e27228)

Figure S1. Map of Wytham Woods. Paired plots (ash dominated and non-ash dominated) are nestled within five study locations. Each point represents a plot corner.


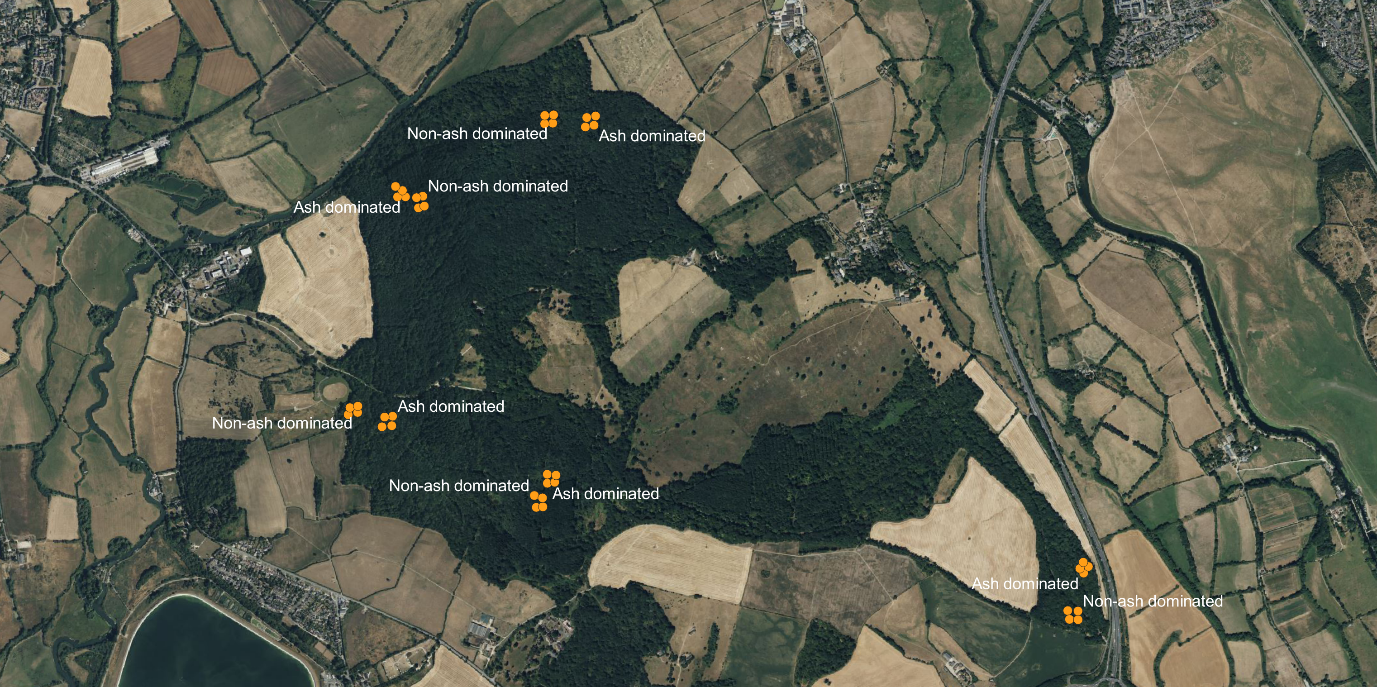

Supplement: Multimedia component 1 [file mmc1.docx]
