# Supplementary material for: Large invertebrate decomposers contribute to faster leaf litter decomposition in Fraxinus excelsior-dominated habitats: Implications of ash dieback
Source: Heliyon. 2024 Mar 5;10(5):e27228. doi: 10.1016/j.heliyon.2024.e27228 (PMC10943353; doi:10.1016/j.heliyon.2024.e27228)

Figure S2. Layout of the mesh bags in each plot. Three clusters of mesh bags were placed in each plot with 24 bags in each cluster and 72 bags in each plot.
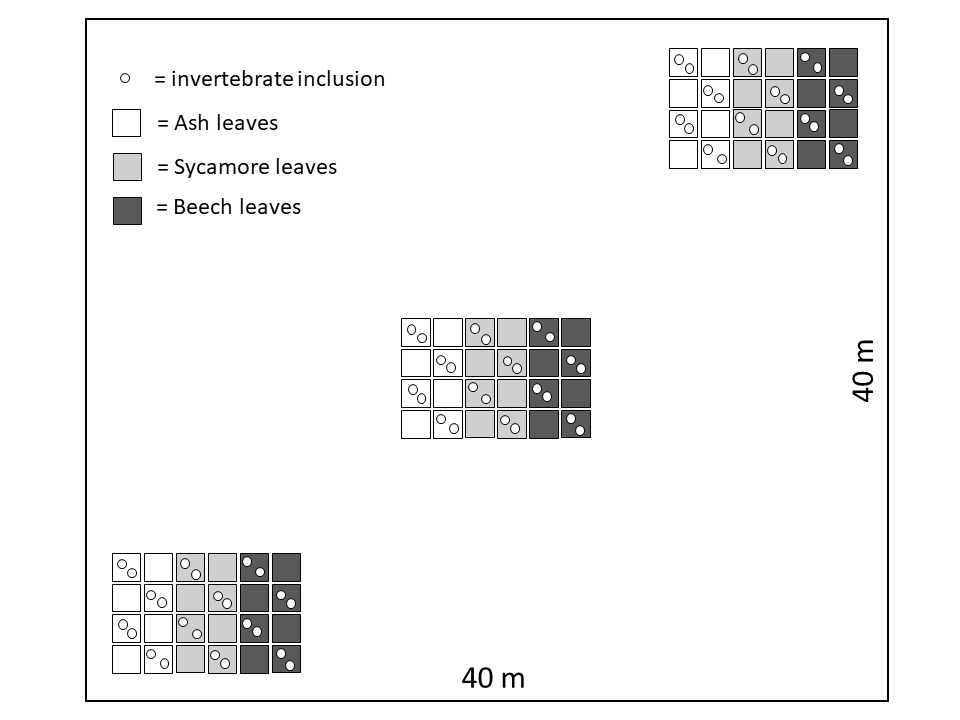

Supplement: Multimedia component 2 [file mmc2.docx]
