# Supplementary figures and images for: Large invertebrate decomposers contribute to faster leaf litter decomposition in Fraxinus excelsior-dominated habitats: Implications of ash dieback
Source: Heliyon. 2024 Mar 5;10(5):e27228. doi: 10.1016/j.heliyon.2024.e27228 (PMC10943353; doi:10.1016/j.heliyon.2024.e27228)

Figure S4. Handling effect (mean  $\pm$  SE) of mesh bags with holes for each leaf species.

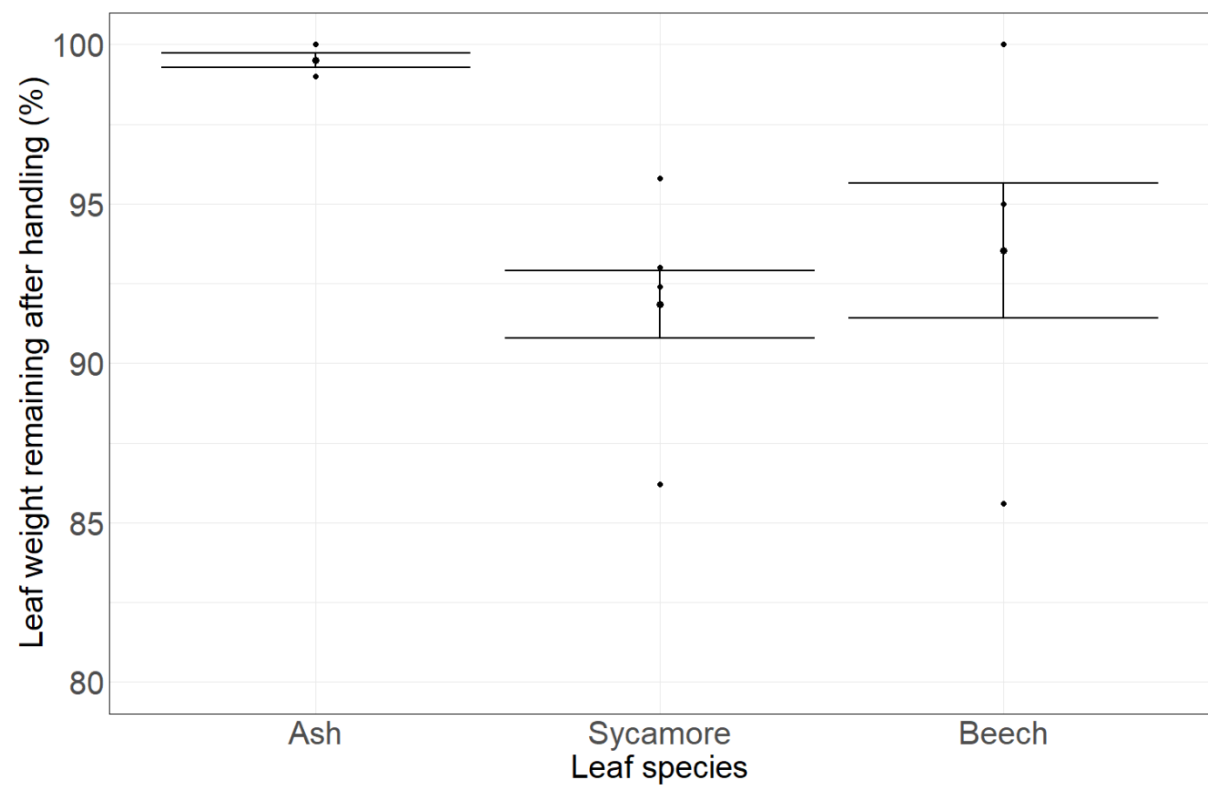

Supplement: Multimedia component 3 [file mmc3.pdf]
