# Supplementary material for: Large invertebrate decomposers contribute to faster leaf litter decomposition in Fraxinus excelsior-dominated habitats: Implications of ash dieback
Source: Heliyon. 2024 Mar 5;10(5):e27228. doi: 10.1016/j.heliyon.2024.e27228 (PMC10943353; doi:10.1016/j.heliyon.2024.e27228)

Figure S5. Monthly dry food intake based on macrofauna biomass collected four times, once every season (February, May, August, November). + indicates medium to large effect sizes (>0.5). None of the paired relationships between habitat types were significant, however, for November, February, and August there were not enough millipedes in the non-ash habitat to enable a statistical analysis.


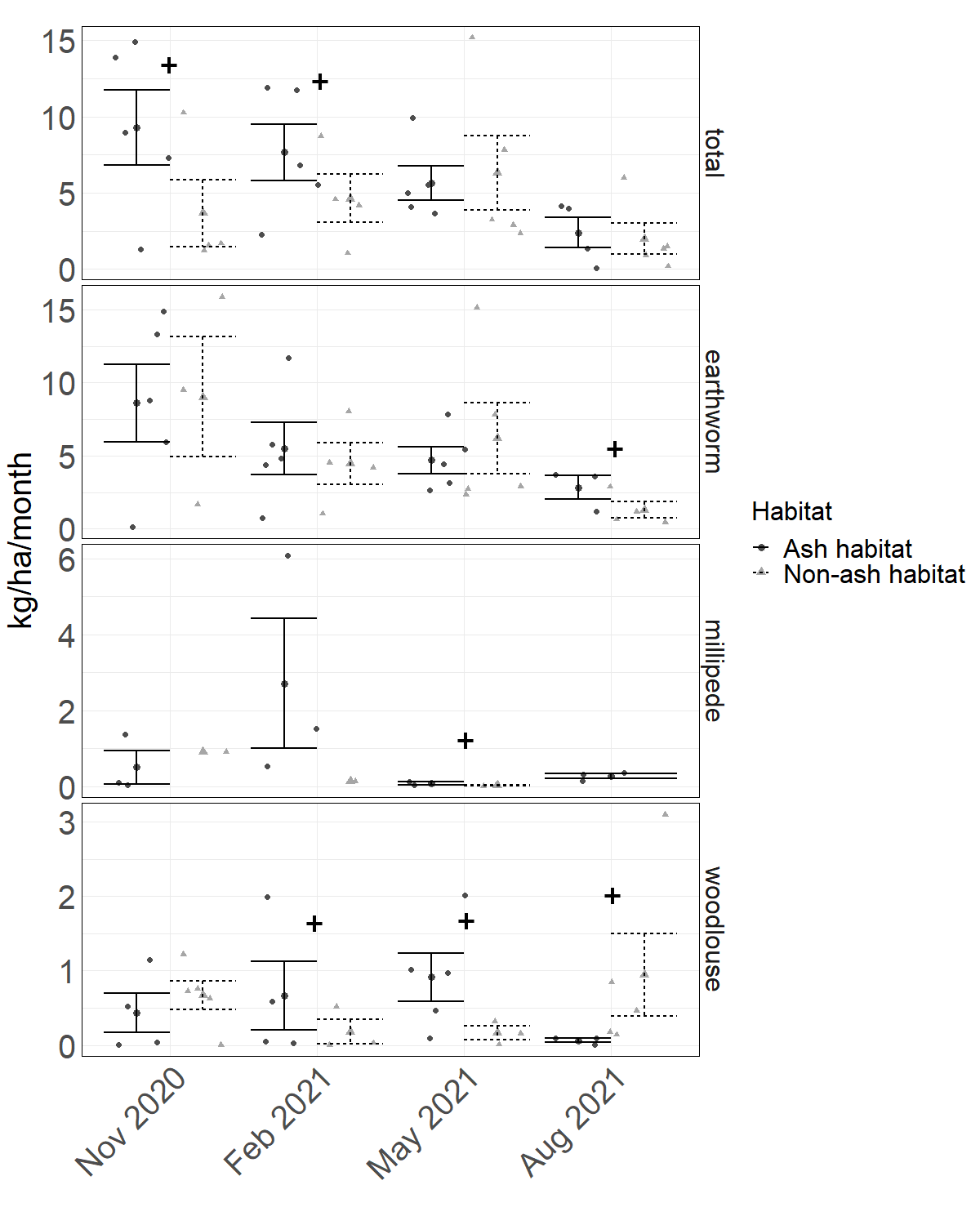

Supplement: Multimedia component 4 [file mmc4.docx]
