# Supplementary material for: Large invertebrate decomposers contribute to faster leaf litter decomposition in Fraxinus excelsior-dominated habitats: Implications of ash dieback
Source: Heliyon. 2024 Mar 5;10(5):e27228. doi: 10.1016/j.heliyon.2024.e27228 (PMC10943353; doi:10.1016/j.heliyon.2024.e27228)

Figure S5. Leaf moisture (mean ± SE) for ash, sycamore and beech in ash dominated and non-ash dominated habitats at each of the four sampling retrievals at 6, 12, 24 and 48 weeks after the start of the experiment. None of the paired relationships were significant using chi square means.


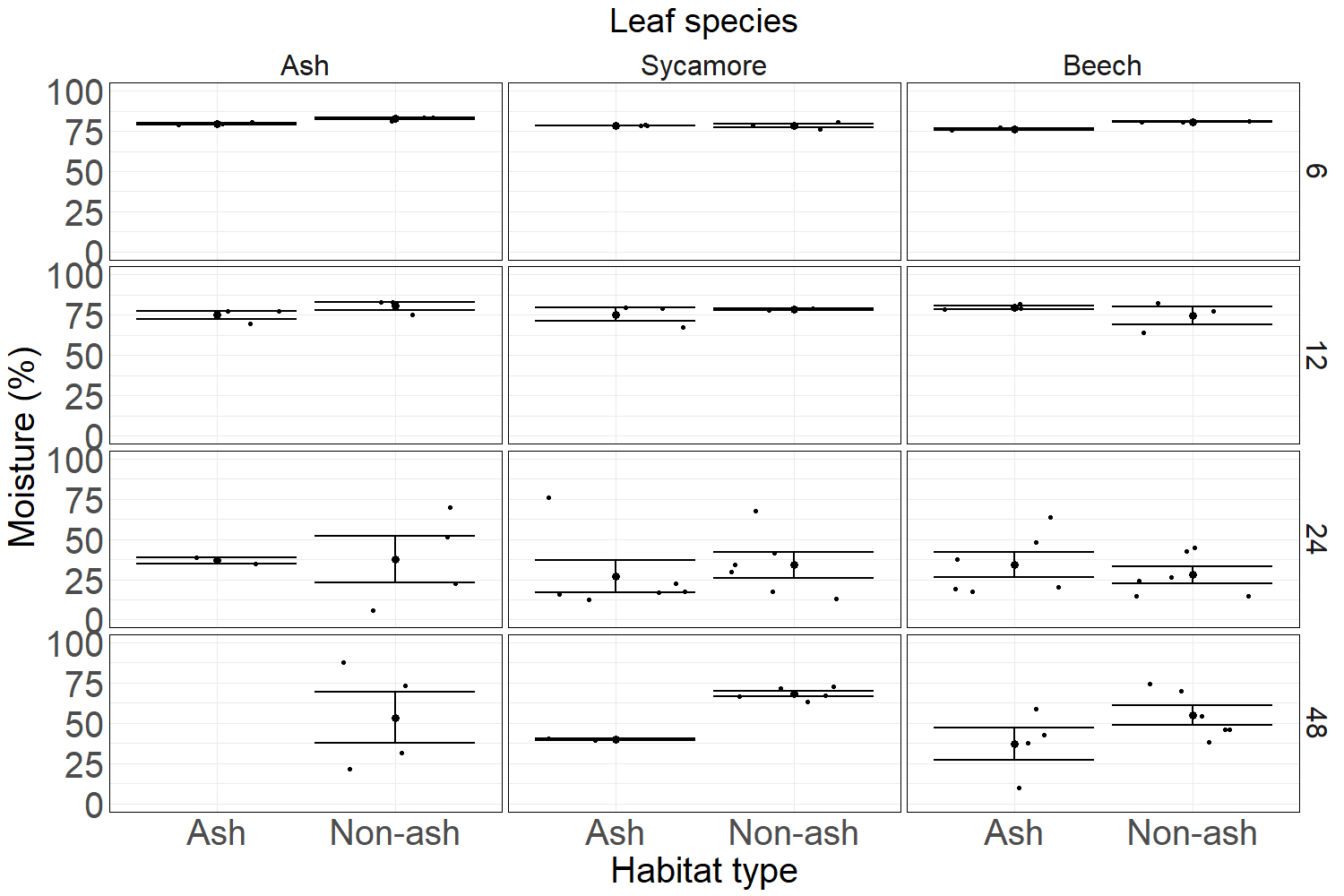

Supplement: Multimedia component 5 [file mmc5.docx]
